# Psychiatric morbidity following liothyronine exposure in autoimmune hypothyroidism: a Swedish nationwide cohort study

**DOI:** 10.1210/jendso/bvag107

**Published:** 2026-05-07

**Authors:** Fredric Hedberg, Jonatan D Lindh, Buster Mannheimer, Tereza Planck, Jakob Skov, Henrik Falhammar, Jan Calissendorff

**Affiliations:** Department of Molecular Medicine and Surgery, Karolinska Institutet, Karolinska University Hospital L1:00, Stockholm SE-171 76, Sweden; Department of Endocrinology, Karolinska University Hospital, Stockholm 171 76, Sweden; Department of Laboratory Medicine, Division of Clinical Pharmacology, Karolinska Institutet, Stockholm 141 52, Sweden; Department of Clinical Pharmacology, Karolinska University Hospital, Stockholm 141 86, Sweden; Department of Clinical Science and Education at Södersjukhuset, Karolinska Institutet, Stockholm 171 77, Sweden; Department of Internal Medicine, Section Diabetes and Endocrinology, Södersjukhuset, Stockholm 118 83, Sweden; Department of Endocrinology, Skåne University Hospital, Malmö 205 02, Sweden; Department of Clinical Sciences Malmö, Lund University, Lund 221 00, Sweden; Department of Molecular Medicine and Surgery, Karolinska Institutet, Stockholm 171 76, Sweden; Department of Medicine, Karlstad Central Hospital, Karlstad 651 85, Sweden; Department of Molecular Medicine and Surgery, Karolinska Institutet, Karolinska University Hospital L1:00, Stockholm SE-171 76, Sweden; Department of Endocrinology, Karolinska University Hospital, Stockholm 171 76, Sweden; Department of Molecular Medicine and Surgery, Karolinska Institutet, Karolinska University Hospital L1:00, Stockholm SE-171 76, Sweden; Department of Endocrinology, Karolinska University Hospital, Stockholm 171 76, Sweden

**Keywords:** LT3, Hashimoto thyroiditis, combination therapy, mental health, epidemiology

## Abstract

**Context:**

Levothyroxine (LT4) is the primary treatment of hypothyroidism. Despite adequate LT4 substitution some patients experience symptoms and the use of adjunct liothyronine (LT3) has increased. Several trials show no clear advantage of LT4 + LT3, and observational studies report conflicting results.

**Objective:**

We aimed to assess the relationship between initiation of LT3 treatment and psychiatric morbidity in patients with autoimmune hypothyroidism.

**Methods:**

All adults in Sweden with autoimmune hypothyroidism without prior psychiatric morbidity initiated on thyroid hormone replacement between 2006 and 2020 were included. Data were obtained from the National Patient Register and the National Prescribed Drug Register. The risk of any psychiatric morbidity after LT4 vs LT4 + LT3 therapy was estimated using Cox regression models with multivariable adjustments and LT3 as a time-dependent covariate.

**Results:**

The total cohort comprised 184 266 individuals. During follow-up, 5346 (2.9%) were exposed to LT3. Median follow-up on LT4 + LT3 was 2.7 years (IQR 1.1-4.4) and 3.8 years (IQR 1.5-7.3) on LT4. Exposure to LT3 was associated with higher risk of any psychiatric morbidity (aHR 1.43, 95% CI 1.34-1.53, *P* < .001). An association was also found with affective or anxiety morbidity (aHR 1.44, 95% CI 1.35-1.54, *P* < .001) and with psychotic morbidity (aHR 1.46, 95% CI 1.11-1.92, *P* = .0067) after multivariable adjustment.

**Conclusion:**

In autoimmune hypothyroidism LT4 + LT3 treatment was associated with increased risk of psychiatric morbidity. This may reflect a potential LT3 effect or an underlying vulnerability among LT3 users, underscoring the need for further studies to clarify any causal relationship.

Levothyroxine (LT4) remains the primary treatment of hypothyroidism [1]. Nevertheless, it has been estimated that up to 10% of patients with adequate LT4 treatment still experience symptoms [2]. Gene polymorphisms, autoimmunity or awareness of chronic disease have been proposed as tentative explanations [3]. The use of liothyronine (LT3) as an adjunct to LT4 has increased [4, 5] and in Sweden LT3 prescriptions have increased by 833% between 2006 and 2023 [6].

Despite ongoing interest, it remains unclear whether combination therapy with LT3 and LT4 provides superior clinical outcomes compared to LT4 monotherapy. Several systematic reviews and meta-analyses of clinical trials have failed to demonstrate an advantage with LT4 + LT3 combination therapy [7-9]. However, the included studies are limited by small sample sizes, heterogeneous inclusion criteria, varying dose regimens, and follow-up not exceeding 12 months. Despite these limitations, some findings suggest potential benefits for combination therapy related to mental health, overall well-being, and patient-reported preference [8, 9]. To better understand the long-term safety and effectiveness of combination therapy, observational studies have provided important data. A Scottish retrospective study (the TEARS study) with up to 17 years of follow-up found no elevated risk of heart disease or fractures among LT3 users compared to LT4 monotherapy [10], but LT3 was associated with an increased risk of being prescribed antipsychotic medication. A marginally increased risk for breast cancer was noted, an association not replicated in a Swedish register-based study [11]. Notably, a large retrospective study reported a lower risk of dementia and mortality in LT3 treated patients with hypothyroidism compared to LT4-only users [12].

A recent nationwide observational study from our group showed that psychiatric morbidity preceding autoimmune hypothyroidism was associated with subsequent treatment with LT3 [13]. However, this study did not address whether LT3 treatment influenced psychiatric outcomes. Considering this, our aim was to investigate the association between LT3 exposure and subsequent development of psychiatric morbidity among patients with autoimmune hypothyroidism without prior psychiatric morbidity.

## Materials and methods

### Study design and setting

This was a retrospective population-based cohort study, encompassing all individuals residing in Sweden aged 18 years or older between 2005 and 2020.

### Data sources

Data were retrieved from two national registers maintained by the National Board of Health and Welfare: The National Patient Register (NPR) and the National Prescribed Drug Register (NPDR). The registers were linked at the individual level using the unique personal identity number assigned to all residents in Sweden [14].

The NPR contains documentation on both inpatient and specialist outpatient visits. Data on hospital admissions with nationwide coverage have been available since 1987, and specialist outpatient visits since 2001. Data have been recorded according to the Swedish version of the Tenth Revision of International Classification of Diseases (ICD-10) since 1997. The NPR has an almost complete coverage of the Swedish population and a positive predictive value for diagnoses ranging from 85% to 95% [15]. The NPDR contains information on all pharmacy-dispensed prescriptions, including primary care, in Sweden since July 1, 2005

### Study population

The study included all individuals ≥18 years of age residing in Sweden with at least one dispensed prescription of thyroid hormone replacement therapy, identified by Anatomical Therapeutic Chemical (ATC) code H03A between July 1, 2005, and December 31, 2020.

To ensure a clinically homogeneous study population and to minimize confounding from heterogeneous etiologies of hypothyroidism the analyses were restricted to individuals with autoimmune hypothyroidism. Autoimmune hypothyroidism was defined by excluding other known causes of hypothyroidism based on ICD-10 codes recorded between 1997 and 2020, in line with previously applied methods in several Swedish register-based studies [16-18]: congenital hypothyroidism (E030-E031), Graves’ disease (E050), thyroid cancer (C73), pituitary dysfunction, eg, central hypothyroidism (E230-E237), and postsurgical and radioiodine-induced hypothyroidism (E890).

Patients prescribed amiodarone or lithium—medications known to affect thyroid function, at any point between 2005 and 2020, were also excluded. Information on multikinase inhibitors, immune checkpoint inhibitors, or interferon treatment was not available in this study.

After exclusion of identifiable nonautoimmune causes, the remaining cohort is expected to predominantly reflect autoimmune hypothyroidism in routine Swedish clinical practice.

Index date was defined as the dispensation date of the first prescription of any thyroid hormone substitution therapy (ATC H03A), regardless of type. To ensure the inclusion of only incident users, patients with any dispensation of thyroid hormone prior to July 1, 2006, were excluded. Hence, all included individuals initiated on LT4 after this date contributed person-time as unexposed until any subsequent LT3 use.

Patients with documented psychiatric diagnoses or psychiatric medication use before the index date were excluded. Psychiatric morbidity was defined as ICD-10 codes F00-F99, excluding F7 (intellectual disabilities) and F8 (developmental disorders), or at least one dispensation of a psychiatric medication (ATC codes N05 or N06A, excluding N05AB01, N05AB04, and N06AA).

Follow-up continued until the occurrence of the outcome, death or the end of the study period, whichever came first.

Diagnostic and medication codes (ICD-10 and ATC) used are provided in Table 1.

**Table 1:** ICD-10 and ATC codes used in the study

Definition	Details	Excluded codes	Details of excluded codes
Thyroid Hormone Therapy	H03A: thyroid hormones, H03AA01: levothyroxine, H03AA02: liothyronine, H03AA03: levothyroxine +liothyronine, H03AA04: tiratricol*^a^*, H03AA05: desiccated thyroid extract*^a^*	—	—
Psychiatric Morbidity	ICD-10 F00-F99: all psychiatric diagnoses	F7, F8	F7: intellectual disabilities, F8: developmental speech/language disorders
	ATC N05, N06A: psychiatric medications	N05AB01, N05AB04, N06AA	N05AB01: dixyrazine, N05AB04: prochlorperazine, N06AA: nonselective monoamine reuptake inhibitors (Tricyclic Antidepressants)
Affective or Anxiety Morbidity	ICD-10 F3, F4: affective and anxiety diagnoses	—	—
	ATC N05AN, N05B, N05C, N06A: medications for affective/anxiety disorders	N06AA	N06AA: nonselective monoamine reuptake inhibitors (Tricyclic Antidepressants)
Psychotic Morbidity	ICD-10 F2: psychotic diagnoses	—	—
	ATC N05A: medications for psychotic disorders	N05AA02, N05AB01, N05AB04, N05AN	N05AA02: levomepromazine, N05AB01: dixyrazine, N05AB04: prochlorperazine, N05AN: lithium
Exclusion—Causes of Hypothyroidism		ICD-10: E030, E031, E050, C73, E230-E237, E890	E030-E031: congenital hypothyroidism, E050: graves’ disease, C73: thyroid cancer, E230-E237: pituitary dysfunction (eg, central hypothyroidism), E890: postsurgical and radioiodine-induced hypothyroidism
Exclusion—Medication affecting thyroid function		ATC: C01BD01, N05AN01	C01BD01: amiodarone, N05AN01: lithium

^*a*^Not registered in Sweden, available only by special license.

To assess robustness, we performed two sensitivity analyses. In the first analysis, we restricted the cohort to individuals with evidence of 12 months LT4 treatment, defined as at least three dispensations on separate dates during the first year after treatment initiation. This restriction was applied to reduce misclassification of transient LT4 users, for example individuals treated temporarily during pregnancy or following destructive thyroiditis. To avoid using postindex information to define eligibility, follow-up was initiated 12 months after the first LT4 dispensation among those fulfilling the run-in criterion. In an additional sensitivity analysis, we applied a 90-day lag after initiation of LT3 treatment. This approach was used to reduce the risk that psychiatric outcomes occurring shortly after treatment switch were attributed to LT3 exposure, as such events may reflect preexisting symptoms or clinical deterioration leading to the treatment change.

### Exposure

The exposure of interest was defined as the dispensation of LT3, identified using ATC codes H03AA02 or H03AA03, at any time during the follow-up period. LT3 exposure was treated as a time-dependent variable, meaning individuals contributed with person-time to the unexposed group (LT4 treatment) until the first dispensation of LT3, after which they contributed to the exposed group regardless of whether LT4 was continued. Individuals continuing LT4 monotherapy throughout follow-up contributed only to the unexposed group. In Sweden, LT3 is almost exclusively prescribed in combination with LT4. Thus, the exposed group represents LT4 + LT3 combination therapy. Desiccated thyroid extract (DTE) is available only through a special license, and DTE users were not part of the study cohort.

### Outcomes

The primary outcome was incident psychiatric morbidity, defined as either a recorded diagnosis corresponding to ICD-10 codes F00-F99, excluding F7 and F8, or a dispensation of psychiatric medication with ATC codes N05 or N06A, excluding N05AB01, N05AB04, and N06AA (Table 1).

Two secondary outcomes defined a priori were also examined: affective or anxiety morbidity, defined as ICD-10 codes F3 (mood disorders) or F4 (neurotic, stress-related, and somatoform disorders), or dispensation of medications under ATC codes N05AN, N05B, N05C, and N06A (excluding N06AA).

Psychotic morbidity was defined as ATC code F2 (schizophrenia, schizotypal, and delusional disorders), or dispensation of antipsychotics (ATC N05A, excluding N05AA02, N05AB01, N05AB04, and N05AN).

### Statistical analyses

Continuous variables were presented as medians with interquartile ranges (IQR), and categorical variables were reported as frequencies (*n*) and percentages (%). Differences between groups were assessed using the χ^2^ test for categorical variables and the Mann–Whitney U test for continuous variables.

We used Cox proportional hazards regression models to estimate the association between LT3 treatment and psychiatric morbidity. LT3 exposure was treated as a time-dependent covariate to account for variation in timing of treatment initiation during follow-up [19].

Separate models were fitted for the primary outcome (psychiatric morbidity) and for secondary outcomes (affective or anxiety morbidity, and psychotic morbidity).

All models were adjusted for age (18-29, 30-49, 50-74, and ≥75 years), sex, county of residence, and index year. Age was modeled categorically to account for potential nonlinear associations with LT3 treatment initiation and psychiatric morbidity. To account for intra-individual correlation, we used a cluster variable based on the unique pseudonymized identifier assigned by the National Board of Health and Welfare.

Results were presented as hazard ratios (HR) and adjusted hazard ratios (aHR) with 95% confidence intervals (CI). Statistical significance was defined as *P* < .05. All analyses were conducted using R (version 4.3.1), including packages data.table and survival.

### Ethical approval

This study was performed in accordance with the principles of the Declaration of Helsinki and approved by the Swedish Ethical Review Authority. Due to the retrospective nature of the study informed consent was waived.

## Results

### Baseline characteristics

**Figure 1:**
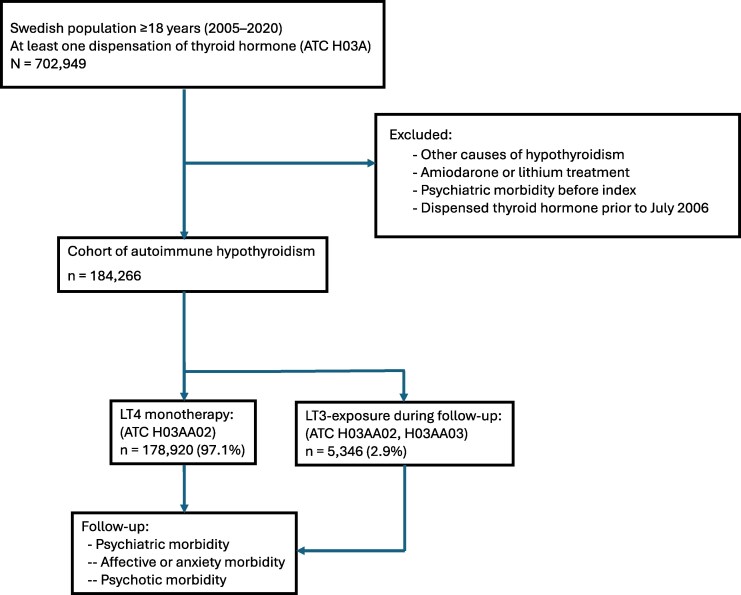
Flowchart of the study cohort. Abbreviations: ATC, anatomical therapeutic chemical; LT4, levothyroxine; LT3, liothyronine.

The total cohort with autoimmune hypothyroidism and newly initiated thyroid hormone replacement therapy comprised 184 266 individuals (Fig. 1). During follow-up, 5346 (2.9%) were subsequently exposed to LT3 and 178 920 (97.1%) remained on LT4 monotherapy. For the total cohort the median age at index was 47 years (IQR 33-65), and 77.8% were women. Among LT3-exposed individuals, the median age at index was 41 years (IQR 32-50) and 88.5% were women, compared with a median age of 48 years (IQR 33-65) and 78.4% women in the unexposed group only treated with LT4. For the LT3-exposed group the median inclusion date was October 2011 (IQR April 2009-November 2014) and March 2013 (IQR December 2, 2009-August 2016) for the unexposed LT4 monotherapy group (Table 2).

**Table 2:** Characteristics at index of the study cohort with autoimmune hypothyroidism between 2006 and 2020

	LT3 users n = 5346 (2.9%)	LT4-only users n = 178 920 (97.1%)
Female n (%)	4733 (88.5)	140 262 (78.4)
Age years median (IQR)	41 (32-50)	48 (33-65)
Median inclusion date (IQR)	Oct 2011 (Apr 2009-Nov 2014)	Mar 2013 (Dec 2009-Aug 2016)

Abbreviations: IQR, interquartile range; LT3, liothyronine; LT4, levothyroxine.

### Follow-up time

The median follow-up time after exposure to LT3 treatment was 2.7 years (IQR 1.1-4.4) compared with 3.8 years (IQR 1.5-7.3) while on LT4 monotherapy. Patients who switched from LT4 to LT4 + LT3 or LT3 treatment contributed follow-up time to both categories, depending on treatment period.

### LT3 treatment and psychiatric morbidity

During follow-up, 53 431 incident psychiatric events occurred. In unadjusted models LT3 exposure was associated with a 34% increased risk of any psychiatric morbidity (HR 1.34, 95% CI 1.26-1.43, *P* < .001) and a 38% increased risk of affective or anxiety morbidity (HR 1.38, 95% CI 1.30-1.48, *P* < .001). No association was observed for psychotic morbidity (HR 0.92, 95% CI 0.71-1.21, *P* = .57) (Table 3).

**Table 3:** Hazard ratios for psychiatric morbidity among patients with autoimmune hypothyroidism treated with liothyronine and levothyroxine (LT3 + LT4) in combination or LT3 vs levothyroxine monotherapy (LT4) between 2006 and 2020

Outcome	Number of events	Hazard ratio (95% CI)	*P*-value (HR)	Adjusted HR (95% CI)	*P*-value (aHR)
Primary analysis:					
Psychiatric morbidity	53 431	1.34 (1.26-1.43)	<.001	1.43 (1.34-1.53)*^a^*	<.001
Men	11 233	1.37 (1.15-1.64)	<.001	1.56 (1,31-1.87)*^b^*	<.001
Women	42 198	1.33 (1.24-1.42)	<.001	1.40 (1.31-1.50)*^b^*	<.001
<30 years	7707	1.53 (1.31-1.77)	<.001	1.52 (1.31-1.77)*^c^*	<.001
30-49 years	17 842	1.39 (1.26-1.52)	<.001	1.38 (1.26-1.51)*^c^*	<.001
50-74 years	18 460	1.45 (1.29-1.64)	<.001	1.44 (1.28-1.63)*^c^*	<.001
≥75 years	9422	1.10 (0.71-1.70)	.68	1.09 (0.71-1.69)*^c^*	.70
Secondary analyses:					
Affective or anxiety morbidity	50 726	1.38 (1.30-1.48)	<.001	1.44 (1.35-1.54)*^a^*	<.001
Men	10 182	1.50 (1.26-1.79)	<.001	1.67 (1.40-2.00)*^b^*	<.001
Women	40 544	1.35 (1.26-1.45)	<.001	1.40 (1.31-1.51)*^b^*	<.001
<30 years	7456	1.49 (1.28-1.74)	<.001	1.49 (1.28-1.74)*^c^*	<.001
30-49 years	17 448	1.39 (1.26-1.51)	<.001	1.38 (1.25-1.51)*^c^*	<.001
50-74 years	17 509	1.54 (1.36-1.73)	<.001	1.52 (1.34-1.71)*^c^*	<.001
≥75 years	8313	1.20 (0.76-1.88)	.43	1.20 (0.76-1.91)*^c^*	.44
Psychotic morbidity	3022	0.92 (0.71-1.21)	.57	1.46 (1.11-1.92)*^a^*	.0067
Men	930	1.05 (0.58-1.91)	.86	1.69 (0.92-3.08)*^b^*	.088
Women	2092	0.95 (0.70-1.28)	.74	1.42 (1.05-1.93)*^b^*	.024
<30 years	336	1.88 (1.32-3.11)	.015	1.91 (1.15-3.18)*^c^*	.012
30-49 years	521	1.28 (0.81-2.03)	.30	1.34 (0.84-2.14)*^c^*	.22
50-74 years	850	1.38 (0.84-2.27)	.20	1.53 (0.93-2.52)*^c^*	.093
≥75 years	1315	0.97 (0.31-3.02)	.96	1.11 (0.36-3.46)*^c^*	.85

^*a*^Adjusted for age, sex, region, and index year;

^*b*^Adjusted for age, region, and index year;

^*c*^Adjusted for sex, region, and index year.

After multivariable adjustment, LT3 exposure remained associated with a 43% higher risk of any psychiatric morbidity (aHR 1.43, 95% CI 1.34-1.53, *P* < .001) and a 44% higher risk of affective or anxiety morbidity (aHR 1.44, 95% CI 1.35-1.54, *P* < .001). The adjusted model also revealed an association with psychotic morbidity (aHR 1.46, 95% CI 1.11-1.92, *P* = .0067) (Table 3).

Results for aHR across the stratified analyses for sex and age groups <30, 30-49, and 50-74 years were broadly consistent with the main models for any psychiatric morbidity and affective or anxiety morbidity (psychiatric morbidity aHR1.38-1.56 and affective or anxiety morbidity aHR 1.38-1.67). In the oldest age group (≥75 years), no association was observed in any of the stratified analyses. For psychotic morbidity, an increased risk associated with LT3 exposure was seen only among women and individuals younger than 30 years (aHR 1.42, *P* = .024 and 1.91, *P* = .012, respectively) (Table 3).

### Sensitivity analyses

In sensitivity analysis 1, restricting the cohort to individuals with at least 12 months of LT4 treatment, 3847 individuals (2.8%) switched to adjunct LT3 during follow-up, while 134 400 (97.2%) remained on LT4 monotherapy. After multivariable adjustment for age, sex, region, and index year, LT3 treatment remained associated with an increased risk of any psychiatric morbidity (aHR 1.37, 95% CI 1.26-1.49, *P* < .001), affective or anxiety morbidity (aHR 1.39, 95% CI 1.27-1.50, *P* < .001), and psychotic morbidity (aHR 1.54, 95% CI 1.09-2.16, *P* = .013).

In sensitivity analysis 2, where a 90-day lag after LT3 initiation was applied, results were similar. After adjustment, LT3 treatment was associated with an increased risk of any psychiatric morbidity (aHR 1.40, 95% CI 1.31-1.50, *P* < .001), affective or anxiety morbidity (aHR 1.41, 95% CI 1.32-1.51, *P* < .001), and psychotic morbidity (aHR 1.47, 95% CI 1.11-1.94, *P* = .0065).

## Discussion

In this nationwide retrospective cohort study of patients with autoimmune hypothyroidism, LT3 exposure was associated with an increased risk of incident psychiatric morbidity. The excess risk was consistent both for the composite outcome of any psychiatric morbidity and for affective or anxiety morbidity. These results remained robust after multivariable adjustment and were broadly consistent in stratified analyses by sex and across the age groups <30, 30-49, and 50-74 years. For incident psychotic morbidity, no association was observed in the crude analysis, but after multivariable adjustment, an increased risk emerged. However, given the wider CIs, this finding should be interpreted with caution. This shift in association for psychotic morbidity following adjustment suggests that differences in the age and sex distributions, as well as temporal patterns, acted as confounders in the crude model. However, the adjusted association was not uniform across all strata and appeared to be driven by women and individuals younger than 30 years.

After restricting the cohort to individuals with 12 months sustained LT4 treatment and applying a 90-day lag after LT3 initiation, effect estimates remained broadly similar to the main analyses, although slightly attenuated for any psychiatric morbidity and affective or anxiety morbidity. The restriction to sustained LT4 use likely reduced misclassification of low-risk transient LT4 users, while the lagged exposure analysis minimized the influence of early events following treatment change. The persistence of the association across analyses supports the robustness of the findings.

Several clinical trials have evaluated the effects of LT4 + LT3 combination compared with LT4 monotherapy. The majority reported no additional benefits of combination therapy [20]. However, a randomized double-blind cross-over trial by Nygaard et al [21] demonstrated improvements in quality-of-life and depression scores in LT4 + LT3 treated patients with autoimmune hypothyroidism. In addition, a small clinical trial by Bunevicius et al [22] that also included patients with thyroid cancer, reported improvements on anxiety and depression scores on combination therapy. These results contrast with a randomized controlled trial by Walsh et al, that showed no benefit of combined LT4 + LT3 therapy compared with LT4 alone on quality of life, psychological well-being, thyroid-related symptoms, and cognitive function [23]. Instead, General Health Questionnaire (GHQ-28) total and social dysfunction scores were worse, and VAS ratings indicated more anxiety during combination therapy.

Two large retrospective cohort studies evaluating the effects of combination therapy included psychiatric outcomes [10, 24]. The TEARS study, comprising 400 LT3 users and 33 955 LT4-only users, showed no increased risk of mental health disorders or being prescribed antidepressant medication among LT3 users, although an increased risk of being prescribed antipsychotic medication was observed. This pattern may indicate a more subtle increase in psychiatric morbidity, with symptoms sufficiently severe to prompt pharmacologic treatment but not consistently captured as formal diagnoses. The study by Yi et al [24] with 1887 LT3 users and 30 303 LT4-only users, found no differences in the risks of mood or anxiety disorders between the treatment groups in the total cohort of patients with hypothyroidism. Stratified analyses on treatment duration suggested a lower risk of both outcomes with short-term LT3 use (<52 weeks) and no differences with long-term use (≥52 weeks). The observed protective association with short-term LT3 use may reflect an initial symptomatic improvement that disappears over time or a placebo effect. In the subgroup without thyroid cancer, a lower risk of mood disorders was observed for LT3 users. Stratified analyses in this subgroup, showed no differences for either mood or anxiety disorders with treatment duration <52 or ≥52 weeks, which may be due to type II error from smaller sample sizes. Notably, these observational studies did not consider LT4 monotherapy preceding adjunct LT3 treatment, which may have introduced immortal time and lag time bias possibly favoring LT3 treatment. This was accounted for in our study by using a time-dependent covariate for LT3 treatment.

Prior work has shown that patients diagnosed with hypothyroidism and treated with LT4 have an increased risk of subsequently being diagnosed with a psychiatric disorder or receiving antidepressant or anxiolytic treatments [25]. Additionally, a diagnosis of hyperthyroidism has been associated with excess risk of hospitalization for psychiatric disorders and treatment with antipsychotics, antidepressants, and anxiolytics [26].

A potential mechanism for the observed risk of psychiatric outcomes in our study may be related to the pharmacokinetics of LT3. Due to its short half-life, LT3 produces serum T3 fluctuations with peaks above the reference range [27], which may provoke symptoms of hyperthyroidism such as anxiety, irritability, and mood disturbances. An alternative explanation is that patients with greater underlying psychiatric vulnerability or emerging psychiatric symptoms were more likely to receive LT3, thereby showing an increased incidence of psychiatric diagnoses after initiation. Evidence from the study by Thvilum et al [25] suggests that hypothyroidism is associated with an increased risk of subsequent psychiatric morbidity, making a strong protective effect of LT4 treatment unlikely.

Somatic comorbidity differs across age groups, with younger adults typically having fewer chronic conditions and older individuals having a higher disease burden. The broadly similar estimates across the age groups <30 up to 74 years for any psychiatric morbidity and affective or anxiety morbidity suggest that age-specific comorbidity patterns alone are unlikely to account for the findings. However, subclinical psychiatric vulnerability or symptom burden preceding LT3 initiation may still contribute across all age strata. In the oldest patients (≥75 years), a higher burden of comorbidity may lead clinicians to avoid LT3 treatment [28], resulting in a healthier subset receiving LT3 which may account for the absence of any detectable differences in psychiatric morbidity compared with LT4 monotherapy. Also, competing risks, including shorter survival time may limit the possibility for a psychiatric diagnosis or treatment to be recorded in this age group.

The main strengths of our study include its large, nationwide cohort of a homogeneous population of autoimmune hypothyroidism and the use of real-world data. The time-dependent exposure definition allowed us to account for changes in treatment during follow-up, thereby reducing the risk of exposure misclassification and avoiding immortal time bias. The consistency of results across the sensitivity analyses supports the robustness of the findings.

However, several limitations should be noted. We lacked information on laboratory data, prescribed doses and duration of LT3 exposure, as well as detailed data on comorbidities, socioeconomic factors, and treatment decisions, which limits the interpretation. The retrospective observational design does not allow causal inference, and despite adjustment for key covariates, residual confounding cannot be ruled out. Surveillance bias and protopathic bias may also have influenced the observed associations. Although a lag-period analysis was applied to reduce potential protopathic bias, some residual reverse causation cannot be excluded. Neither did we have data on the use of multikinase inhibitors, immune checkpoint inhibitors or interferon treatment nor information on genetic variants in deiodinases or thyroid hormone transporters. Despite performing a sensitivity analysis requiring 12 months of sustained LT4 treatment, some residual misclassification of autoimmune hypothyroidism may remain.

In summary, this study provides real-world evidence from a nationwide cohort that LT3 treatment in autoimmune hypothyroidism is associated with an increased risk of developing psychiatric morbidity, particularly affective or anxiety morbidity and possibly psychotic morbidity. The observed associations may reflect underlying vulnerability among patients who are prescribed LT3, including the possibility that LT3 may be initiated in response to emerging symptoms of poor mental well-being rather than being causally related to psychiatric morbidity. Despite these limitations, the results remain clinically relevant and highlight the need to better understand the underlying mechanisms and relationship between LT3 and psychiatric outcomes.

Adequately powered randomized controlled trials with longer follow-up periods including validated psychiatric rating scales are needed to confirm these findings and to provide more robust evidence on the causal effects of LT3 treatment on psychiatric outcomes.

In addition, future prospective observational studies incorporating data on treatment decisions, thyroid hormone concentrations, other causes of hypothyroidism, and genetic variations in deiodinases and hormone transporters are warranted to further explore potential underlying mechanisms.

## Data Availability

The data that support the findings of this study are available from the corresponding author upon reasonable request.
